# Beyond ketosis: the search for the mechanism underlying SGLT2-inhibitor benefit continues

**DOI:** 10.1172/JCI187097

**Published:** 2024-12-16

**Authors:** Justin H. Berger, Brian N. Finck

**Affiliations:** 1 Division of Pediatric Cardiology, Department of Pediatrics and; 2 Division of Nutritional Science and Obesity Medicine, Department of Medicine, Washington University School of Medicine, St. Louis, Missouri, USA.

## Abstract

Despite the impressive clinical benefits and widespread adoption of sodium glucose cotransporter 2 inhibitors (SGLT2i) to treat all classes of heart failure, their cardiovascular mechanisms of action are poorly understood. Proposed mechanisms range broadly and include enhanced ketogenesis, where the mild ketosis associated with SGLT2i use is presumed to be beneficial. However, in this issue of the *JCI*, carefully conducted metabolic flux studies by Goedeke et al. comparing the effects of SGLT2i and exogenous ketones suggest differential effects. Thus, the mechanisms of action for SGLT2i are likely pleiotropic, and further work is needed to fully understand their beneficial effects.

## SGLT2i as a powerful heart failure therapy

Sodium glucose cotransporter 2 inhibitors (SGLT2i) are perhaps the most powerful pharmaceutical advancement to date in the treatment of heart failure (HF), which makes the serendipitous identification of the cardiovascular benefit of these drugs originally intended to treat diabetes all the more notable. Major clinical trials in over 80,000 adult patients demonstrated an extraordinary 20%–30% reduction in HF hospitalization rates and death in patients with HF with reduced ejection fraction (HFrEF) and hospitalization in those with HF who had preserved ejection fraction (HFpEF) irrespective of comorbid diabetes (1–4). SGLT2i are now the standard of care for HF, with rapid US Food and Drug Administration approval and widespread adoption into US and European clinical HF guidelines (5, 6). Remarkably, the molecular mechanisms by which they mediate their cardiovascular benefits remain poorly understood.

Isolated from apple tree root bark, the lead compound phlorizin was discovered over 150 years ago, but only in the 1990s identified as a direct inhibitor of SGLT1 (which is broadly expressed, including in the heart) and SGLT2 (which is largely restricted to the kidney). Extensive clinical study and use of the gliflozin family derivatives for the treatment of diabetes began in the 2000s. By preventing glucose reabsorption in the kidney and promoting glucosuria, SGLT2i lower blood glucose and their use is associated with modest weight loss and lower systolic blood pressure. However, the observed cardioprotective benefits of SGLT2i in HF are much greater than what similar, established therapeutics (e.g., diuretics, antidiabetic agents) achieve, suggesting unique mechanisms of action.

## Wide-ranging mechanistic hypotheses

Multiple mechanisms for the benefit of SGLT2i in HF have been proposed. Prevailing theories recognize that SGLT2 is not expressed in cardiomyocytes; therefore, the beneficial effects in HF must be off target (not mediated by SGLT2) or due to extracardiac SGLT2 inhibition (e.g., in the kidney). Proposed extracardiac mechanisms include alterations in microbiome-derived toxic intermediates such as p-cresol sulfate (7), improved endothelial function (8), alterations in epicardial adipose tissue mass and function (9, 10), and/or additive benefit of the pleotropic effects, e.g., weight loss largely related to enhanced natriuresis, glucose wasting, and reduced blood pressure (11). Lastly, many labs have focused on potential shifts in myocardial/mitochondrial energetics related to the reproducible mild ketosis, glucose wasting, and subsequent altered hormonal signaling seen with SGLT2i treatment.

Cardiac myocytes are metabolic omnivores that use fatty acid and carbohydrate oxidation to generate ATP in the healthy state. However, several pathologic conditions, including HF, are associated with metabolic inflexibility, shifting toward anaerobic glucose metabolism and ketone oxidation. The necessary role of endogenous ketone oxidation and beneficial role of exogenous ketones in mouse and human HF studies is well established (12–15). Elegant human studies demonstrate that myocardial ketone uptake is proportional to available circulating ketone concentrations (16) and ketone oxidation enzyme expression increases in HF (12, 13). Small clinical trials, primarily out of Europe, have begun to suggest an acute benefit from exogenous ketone administration (17). Less clear is how much of this benefit is due to improved energetics (ketone oxidation to relieve impaired TCA flux) or nonoxidative, systemic effects such as vasodilation, antiinflammatory signaling, antioxidant effects, and more (18).

## A distinction between ketosis and SGLT2i-induced ketosis

**Figure 1:**
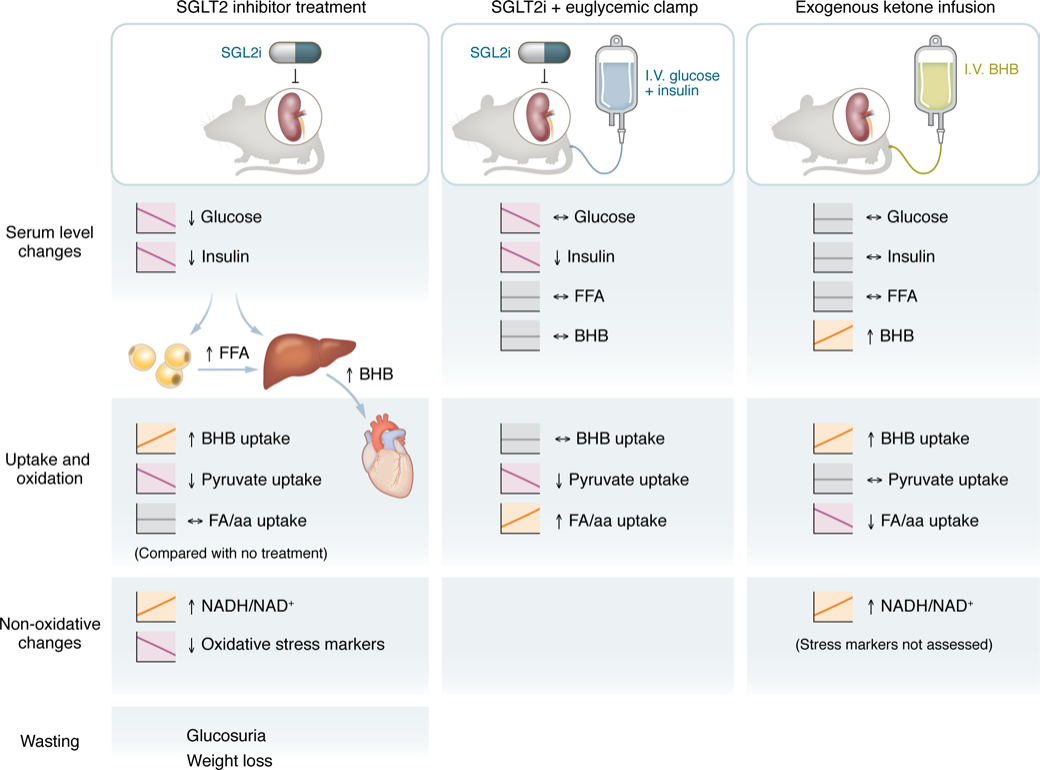
SGLT2i and ketone treatment have overlapping but distinct metabolic consequences. Treatment of nonanesthized rats under three conditions demonstrate differential fuel sources for cardiac oxidation. BHB, beta-hydroxybutyrate; (F)FA (free) fatty acid.

In this issue of the *JCI*, Goedeke and colleagues (19) sought to address this latter hypothesis of myocardial substrate utilization by teasing apart the independent effects of SGLT2i therapy (with its expected ketosis) from exogenous ketone administration in normal rats and in a rat model of myocardial infarction. They utilized a rigorous approach of infusing stable isotope-labeled glucose and β-hydroxybutyrate (BHB) coupled with tandem chromatographic and spectroscopic techniques, previously used to assess whole body substrate metabolism. This method allowed for in vivo measurements of substrate flux in nonanesthetized animals. Acute treatment with the SGLT2i dapagliflozin at clinically relevant doses reliably mirrored the known human clinical effects: glucose wasting, decreased insulin concentrations, increased circulating free fatty acids indicative of lipolysis, and ketosis (Figure 1). Somewhat surprisingly, rats demonstrated weight loss following a single dose of dapagliflozin, which is likely attributed to enhanced natriuresis/diuresis. In this changing neurohormonal and nutrient milieu, tracer studies demonstrated that SGLT2i treatment resulted in a 50% increase in myocardial ketone oxidation, balanced with a reduction in pyruvate oxidation (Figure 1).

Comparing the effects of acute BHB administration and SGLT2i treatment in the context of a euglycemic glucose clamp, Goedeke et al. reconfirmed that myocardial ketone uptake and oxidation were dependent on circulating BHB concentrations. Moreover, this technique also uncovered the most important difference in shifting myocardial energetics between the two study arms. By providing glucose, which prevented the reduction in insulin and suppressed lipolysis, less ketogenesis and ketone oxidation was observed, which blunted the effect on pyruvate oxidation. However, with acute BHB administration, glucose homeostasis was unperturbed, and so ketone oxidation increased (at the expense of fatty acid oxidation). These observations held true in a rat model of ischemic HFrEF where dapagliflozin treatment increased ketone oxidation and decreased pyruvate oxidation, with improved echocardiographic parameters in the SGLT2i cohort. Lastly, findings of improved redox state and decreased oxidative stress markers were similar between exogenous ketone and SGLT2i treatment.

Taken together, these findings clarify mechanisms by which the benefit of SGLT2i may act through ketones as opposed to another unique mechanism. Prior studies have addressed whether pharmacologic induction of ketosis by SGLT2i benefited cardiac function with somewhat conflicting results. Wu et al. demonstrated cardiac benefit from SGLT2i in multiple HFrEF models without observable ketosis (20), while we have shown that reproducible ketosis caused by genetic deletion of SGLT2 is insufficient to recapitulate the cardiac benefit of SGLT2i treatment (21). In cardiometabolic models of HFpEF, exogenous ketone and SGLT2i have rescued the cardiac phenotype to a similar degree via proposed activation of citrate synthase to increase downstream oxidative phosphorylation (22, 23).

## If not ketosis, what else?

Without question, circulating ketones are readily used by the healthy heart and preferentially used by the failing myocardium. Although ketosis has a clear cardiac benefit, ketonemia alone does not appear to recreate the ameliorating effects of SGLT2i. SGLT2i, but not BHB, also enhance lipolysis by reducing circulating insulin concentrations resulting in increased fatty acid oxidation. This effect is only partially reversed with concurrent glucose administration, so this difference may depend on the acute volume status changes (seen as weight loss) and subsequent natriuretic peptide effects on lipolysis (though natriuretic peptides were not assessed). It is also peculiar that expression in ketone oxidation enzymes was not altered in Goedeke et al. (19), which conflicts with multiple published rodent and human reports.

A second key insight from Goedeke et al. (19) highlights the nonoxidative effects of ketones, including changes to redox state and oxidative stress, as well as vasodilation. Indeed, human data suggest that decreased systemic vascular resistance (SVR) is a key feature for acute BHB benefit on cardiac metrics (17). However, prior work relied on cardiac catheterization data, which were unavailable in this study due to the focus on nonanesthetized animals. Was the lack of benefit from acute dapagliflozin administration due to lack of lowered SVR and improved cardiac output? Measuring the acute effects of BHB administration on cardiac metrics (including hemodynamics) in HF for direct comparison to SGLT2i was a missed opportunity.

Moving forward, the field clearly must also consider SGLT2-independent mechanisms of action for SGLT2i. Recent and conclusive genetic evidence has demonstrated that SGLT2i can act independently of SGLT2 to ameliorate HF in mice, since SGLT2i produce beneficial cardiac effects in HFrEF and ischemia models even in germline SGLT2-knockout mice (20, 21, 24). Off-target cardiac candidates include NHE1, a cardiac sodium-hydrogen transporter and a target of cariporide drug trials in the early 2000s (25, 26), PANK1, a rate-limiting enzyme in replenishing CoA pools (27), and other glucose transporters. Inhibition of NHE1 or activation of PANK1, which are expressed in heart, could elicit direct cardiac effects on ionotropic or metabolic signaling that might explain the cardiac benefits of SGLT2i. Thus, the search of the mechanism continues, and will likely require a “Yes, and” understanding of the pleiotropic effects of this drug class.

## Version 1 12/16/2024

Electronic publication
